# Microglia and metastases to the central nervous system: victim, ravager, or something else?

**DOI:** 10.1186/s13046-022-02535-7

**Published:** 2022-11-21

**Authors:** Maria M. Caffarel, Mounia S. Braza

**Affiliations:** 1 Biodonostia Health Research Institute, Basque Country, Spain; 2 Ikarbasque, Basque Foundation for Science, Basque Country, Spain; 3 Department of Oncological Sciences, Icahn School of Medicine at Mount Sinai, New York City, NY USA

## Abstract

Central nervous system (CNS) metastases are a major cause of death in patients with cancer. Tumor cells must survive during their migration and dissemination in various sites and niches. The brain is considered an immunological sanctuary site, and thus the safest place for metastasis establishment. The risk of brain metastases is highest in patients with melanoma, lung, or breast cancers. In the CNS, metastatic cancer cells exploit the activity of different non-tumoral cell types in the brain microenvironment to create a new niche and to support their proliferation and survival. Among these cells, microglia (the brain resident macrophages) display an exceptional role in immune surveillance and tumor clearance. However, upon recruitment to the metastatic site, depending on the microenvironment context and disease conditions, microglia might be turned into tumor-supportive or -unsupportive cells. Recent single-cell ‘omic’ analyses have contributed to clarify microglia functional and spatial heterogeneity during tumor development and metastasis formation in the CNS. This review summarizes findings on microglia heterogeneity from classical studies to the new single-cell omics. We discuss i) how microglia interact with metastatic cancer cells in the unique brain tumor microenvironment; ii) the microglia classical M1-M2 binary concept and its limitations; and iii) single-cell omic findings that help to understand human and mouse microglia heterogeneity (core sensomes) and to describe the multi-context-dependent microglia functions in metastases to the CNS. We then propose ways to exploit microglia plasticity for brain metastasis treatment depending on the microenvironment profile.

## Introduction

Metastasis formation is a complex process in which cancer cells migrate from the primary tumor site and spread to secondary sites. This includes different steps: local invasion (cancer cells invade the area surrounding the primary site), intravasation into the circulation, survival (maintenance in the circulation), extravasation at secondary site(s) (e.g. the central nervous system, CNS), and colonization of secondary site(s) [1]. Only very few metastatic cells will survive throughout all steps of the metastatic cascade due to the host anti-cancer immune response, if this is not controlled by tumor cells [2].

Metastases are becoming a major drawback in the quest to improve the outcome of patients with cancer. In the case of brain metastases, the available treatments (surgery, irradiation/chemotherapy) allow the survival of very few patients for more than 2 years after diagnosis [3]. Metastases to the CNS concern 10 to 50% of patients with tumors, especially patients with lung cancer (40–50%), breast cancer (20–30%), and melanoma (20–25%) [3, 4]. Metastasis (particularly, brain metastasis) biology and regulation by the tumor microenvironment have been elegantly reviewed elsewhere [1, 5].

Tumor-related changes in the expression of genes that increase the cancer cell tropism for a specific organ are frequently correlated to the capacity of cancer cells to overcome specific obstacles (e.g. the blood brain barrier, BBB, in the case of CNS), or to create a permissive niche in an unfavorable environment, with important positive effects on metastasis progression [6, 7]. The CNS is a complex organ in which immune cell entry is limited by the BBB and cerebrospinal fluid barrier, thus creating a perfect environment for a tumor niche [8]. Moreover, the cells (specifically resident immune cells) and tissues of the brain environment significantly influence metastasis progression and contribute to therapy resistance [9]. Tumor-associated macrophages (TAMs), which include microglia and infiltrating macrophages, represent 50% of the total metastatic tumor mass, and are among the cell populations targeted by cancer cells. Their absence significantly impairs the metastatic spread of primary tumors, suggesting that they play an essential role in cancer cell invasion and metastasis formation in the CNS [10, 11]. Microglia are the resident macrophages and the major innate immune cells in the brain. They are of mesodermal origin (from erythromyeloid progenitors present in the yolk sac during embryonic development), and represent a unique cell type among all CNS cells [12–14]. Their high plasticity explains their different roles in function of the specific microenvironmental context (healthy brain or disease) [15]. In healthy brain, they have a crucial role in immune surveillance. Conversely, in disease conditions, they acquire a molecular signature characteristic of their shift from a homeostatic to a disease-associated function [16, 17]. For the successful colonization of the CNS, metastatic tumor cells must engage in a continuous crosstalk with different resident cells (e.g., microglia) to exploit their functions in order to escape the host anti-tumor activity [7]. Depending on the pro- or anti-inflammatory and the spatial–temporal contexts, microglia function might be altered and the crosstalk between them and tumor cells might induce profound changes at the brain metastatic site [18]. Indeed, through early reprogramming during the metastatic process, microglia could be redirected to a pro-invasive and immunosuppressive phenotype to counterattack the anti-tumor immunity and resist to treatment. When established, this tumor-supportive immunosuppressive state might be hard to tackle. Microglia may then support tumor cell progression through the different metastasis steps (from invasion to colonization) and interaction with the metastatic niche [19, 20].

Upon arrival in the brain, metastatic tumor cells promote the recruitment of myeloid cells from secondary lymphoid organs. They also induce a unique microglia molecular signature associated with malignancy. This reprogramming occurs early during brain colonization by cancer cells and is maintained throughout metastasis progression [19]. Therefore, understanding the functional and spatial characteristics of microglia in the metastatic tumor microenvironment is essential for developing novel effective treatments for CNS metastases. In this review, we compared the view on microglia plasticity based on classical studies and on high-resolution ‘omic’ analyses to better understand their role in brain metastases and their impact on this complex multidimensional microenvironment. We also discussed how their function can be modified upon interaction with tumor cells.

## Microglia diversity: the classical concept

**Fig. 1:**
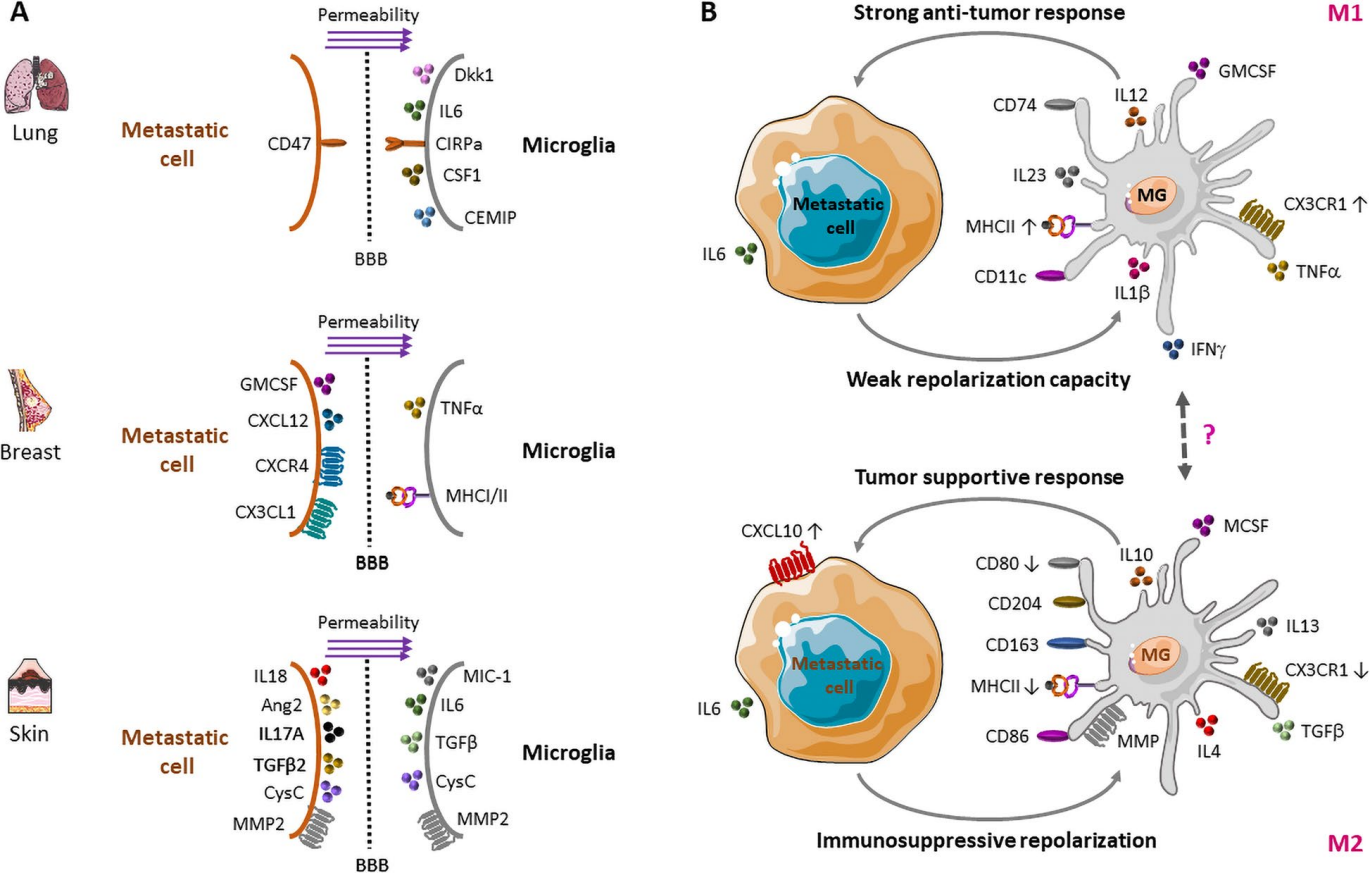
Microglia crosstalk with metastatic cancer cells in the complex brain microenvironment. **A** Melanoma, lung and breast cancers have the highest risk to metastasize to the CNS. These panels summarize the main microglia alterations observed in these metastases to the CNS and their impact on the metastatic cancer cell-microglia cross-talk. **B** Upon cancer cell arrival in the brain, microglial cells will be attracted and recruited to the tumor site. According to the microenvironment context, microglia can differentiate into the M1 pro-inflammatory phenotype (to upregulate pro-inflammatory cytokines and to exert their anti-tumor response against metastatic cells), or into the M2 suppressive phenotype (to upregulate anti-inflammatory cytokines, to promote tumor survival and growth, and to strengthen the metastatic niche). Microglial cells might also present an intermediate M1-M2 phenotype

This section focuses on the main microglia alterations observed in melanoma, lung and breast cancer metastases to the CNS and their impact on metastatic cancer cells and their niche (Fig. 1A).

### Microglia and brain metastases from lung cancer

Few data are available on the immune composition of the microenvironment of brain metastases from lung cancer. Some of these studies used animal models treated with exosomes derived from lung cancer cells and co-culture cell systems to mimic the microglia vascular niche. They demonstrated that a suppressive signal is transferred from the brain endothelium to microglia through the release of endogenous Dickkopf-related protein 1. This causes the switch from the M1 to the M2 phenotype and the gain of pro-tumorigenic features by microglia in the pre-metastatic niche [21]. Among the many factors involved in brain metastases from lung cancer, interleukin (IL)-6 and colony stimulating factor 1 and cell migration-inducing hyaluronidase (CEMIP) are implicated in the repolarization and over-activation of myeloid cells and microglia, respectively, to improve the transmigration of metastatic cancer cells across the BBB and to create a pro-inflammatory metastatic niche [20, 22, 23]. Interestingly, some works showed that human tumor cells escape the microglia immune surveillance by upregulating CD47 and SIRP ⍺ on tumor and microglial cells, respectively (Fig. 1A). This promotes their interaction, reduces microglia phagocytic activity and increases their secretion of trophic factors that might promote the progression of brain metastases from lung cancer [24].

### Microglia and brain metastases from breast cancer

In brain metastases from breast cancer, several immunohistochemistry-based studies have shown a strong expression of granulocyte–macrophage colony-stimulating factor (GM-CSF), C-X-C motif chemokine ligand 12 (CXCL12) and its receptor 4 (CXCR4), and CX3CL1. These factors promote microglial cell proliferation and attraction to the tumor microenvironment, respectively (Fig. 1A). In turn, microglia secrete tumor necrosis factor (TNF) ⍺ that stimulates brain endothelial cells. This increases BBB permeability and immune cell infiltration (including macrophages) that might support the metastatic process [25, 26]. Studies in animal models demonstrated that invading breast cancer cells modulate the microglia activation state and topography. Indeed, microglial cells infiltrate the tumor mass, accumulate in gliosis zones, and are detected in direct contact with tumor cells after extravasation. These microglial cells are heterogeneous, as indicated by the presence of activated (i.e., expressing only major histocompatibility complex (MHC) I, with stellate morphology), hypertrophied (i.e., hypertrophic stellate appearance), and reactive (i.e., expressing MHC I and MHC II, with amoeboid morphology) cells [27]. For instance, in a breast cancer xenograft model, loss of the long non-coding RNA X-inactive specific transcript (*XIST)* reprograms microglial cells toward the suppressive M2-like phenotype to promote brain metastasis formation [28]. This anti-inflammatory M2 phenotype has been detected around the tumor site and is characterized by the expression of arginase-1, mannose receptor 1, inducible nitric oxide synthase, and cyclooxygenase 2 [29].

In addition, gene expression analyses of microglial cells co-cultured with carcinoma cells showed alterations of the Toll-like receptor (TLR), WNT/β-catenin, stromal cell-derived factor 1 (SDF1)-CXCR4, phosphoinositide 3-kinase (PI3K), and chemokine ligand 2/chemokine receptor 2 (CCL2-CCR2) signaling pathways. These signaling cascades are important for the microglia-cancer crosstalk and their inhibition prevents breast cancer metastasis formation and invasion [10, 16].

To better understand the metastatic niche, Simon et al. developed a mouse model to investigate metastatic breast cancer cell-microglia interactions using intravital imaging combined with *ex-vivo* electrophysiology. To this aim, they implanted an optical window on the parietal bone to facilitate cell behavior monitoring in situ in the outer cortex of heterozygous *Cx3cr1*^GFP/+^ mice. After breast cancer cell grafting, they observed a significant accumulation of activated microglia that surrounded the invading tumor cells. The inflammation resulted in significant cortical disorganization and abnormal local field potential spike events at the tumor site [30]. These changes could partly explain the epileptic seizure and cognitive damage observed in patients with brain metastases. Besides tumor cells, microglia can also be modulated by other cell populations of the brain microenvironment. For example, reactive astrocytes with activated signal transducer and activator of transcription (STAT)3 surround metastatic brain lesions and promote the expansion of CD74-expressing microglia that become highly enriched within macro-metastases and produce the growth-promoting factormidkine [31].

### Microglia and brain metastases from melanoma

The communication between metastatic melanoma cells and microglia may influence the secretion of factors that promote vascularization, such as angioprotein-2 (ANG2), by melanoma cells, and of growth differentiation factor 15 (GDF-15, also called macrophage inhibitory cytokine-1, MIC-1) and other pro-inflammatory cytokines by microglia. All these factors promote the metastatic process [32, 33]. Moreover, microglia and melanoma cells reciprocally modulate their gene expression, cell signaling, and cytokine secretion. For instance, melanoma cells significantly affect microglia morphology, proliferation, migration, and matrix metalloprotease (MMP)-2 activation. In turn, microglial cells facilitate phenotypic changes in melanoma cells, resulting in increased proliferation, migration, and MMP-2 activation that promote their aggressiveness [34].

In mouse models of melanoma, IL-17A-STAT3 signaling plays an important role in the metastatic melanoma cell-microglia interaction. IL-17A stimulates angiogenesis and leads to IL-6 synthesis. In turn, IL-6 induces STAT3 activation in melanoma cells that upregulates genes involved in tumor angiogenesis and survival [34, 35]. Moreover, transforming growth factor (TGF)- β expression is increased in microglia, causing tolerance of metastatic melanoma cells by anti-tumor cytotoxic T cells. In a mouse model of melanoma metastases in the brain, TGF- β2 expressed by melanoma cells plays a crucial role in the establishment of metastases specifically in the CNS [36]. In another mouse model of spontaneous melanoma brain metastases, activated microglial cells are recruited to the tumor-brain interface. Depletion of microglia and macrophages using macrophage colony stimulating factor-1 receptor and MMP-3 inhibitors, respectively, drastically reduces the total number and mean size of brain metastases [17]. This suggests that these cell types are key players in melanoma metastases to the brain. Moreover, in vitro and in vivo experiments in immune-deficient mice bearing xenografts of human melanoma brain metastases showed that the extracellular cysteine protease inhibitor cystatin C is involved in the melanoma cell-microglia interaction. Cystatin C secretion is increased in both melanoma and microglial cells. This factor promotes melanoma cell migration and inhibits microglia migration to the melanoma tumor site, thus supporting melanoma brain metastases [37]. Furthermore, pre-clinical data showed an abnormal interaction between microglia and metastatic melanoma cells. This was caused by altered JNK and p38 signaling in microglia that decreases their phagocytic function and supports the metastatic niche [34] (Fig. 1A).

These results in three different cancer types highlight that the interplay between microglia and metastatic cells from melanoma and lung and breast cancer significantly influences the disease outcome. Indeed, microglial cells can have anti- or pro-tumorigenic roles in animal models in function of their activation state and microenvironment. Microglial cells have been detected in human brain metastatic samples, but their association with the clinical outcome is still not clear and requires further validation [38, 39].

Melanoma, lung and breast cancers can also spread in the leptomeninges of brain and spinal cord or in the cerebrospinal fluid, causing leptomeningeal metastases (LM). They concern ~ 23% of patients with melanoma, 9–25% of patients with lung cancer, and 5% of patients with breast cancer, and are associated with very poor survival [4]. Although activated microglia have been found in human samples and mouse models of LM, very little is known about their role in LM development and growth [40, 41].

### The oversimplified M1/M2 microglia concept

Classically, microglial cells have been classified into the M1 and M2 subtypes that have different functions and are induced by different microenvironmental stimuli (Fig. 1B). M1 microglial cells are induced by pro-inflammatory cytokines (e.g., TNFα, interferon gamma, and lipopolysaccharide) [42, 43]. By producing reactive oxygen species that increase STAT1 expression, M1 microglia trigger the secretion of inflammatory cytokines, such as inducible nitric oxide synthase, IL-1β, IL-12 and IL-23. This explains their anti-tumor immune role [44]. Specifically, by expressing MHC II, CD74, CD80/CD86 and CD11c [45], they function as tumor antigen-presenting cells, and activate T cells, leading to tumor cell death and clearance [17, 46, 47]. Conversely, M2 microglial cells are induced by anti-inflammatory cytokines (e.g., IL-4, IL-13, IL-10, and TGFβ) [48]. By overexpressing vascular endothelial growth factor, CD204, CD163, MMPs, and arginase-1 immunomodulatory molecules and releasing immunosuppressive molecules, they promote STAT3 expression and tumor cell growth, and inhibit their antigen presentation function [42, 43, 49, 50] (Fig. 1B).

Activated microglia are an abundant source of inflammatory and anti-inflammatory molecules that can affect tumor progression and also brain metastasis formation. The M2 microglia phenotype results in disturbance of CNS homeostasis. Therefore, M2 cells contribute to support tumor progression and metastasis development, due to the local immunosuppression [28]. Metastatic tumor cells induce the M1 to M2 phenotypic shift that supports their growth. However, it is not clear how they can evade the cytotoxic effect of M1 microglia and what is the exact relationship between M1-M2 microglia balance and tumor metastases during brain invasion by metastatic cancer cells.

Microglia role in CNS diseases, including brain metastases, seems to be complex and goes beyond the oversimplified binary M1-M2 definition or the mixed M1 and M2 functions that cannot explain the myeloid compartment heterogeneity and plasticity. Indeed, when the BBB integrity is affected during tumor progression, the M1-M2 classification is not adequate because of the increase in macrophage number due to the homing of peripheral immune cells in the brain that can enhance the myeloid infiltrate heterogeneity. This makes more difficult to decipher the specific function and phenotype of each subpopulation in this complex microenvironment [51].

### Classical hypotheses to differentiate microglia and infiltrating macrophages

To better understand their specific roles, several studies using human and mouse models tried to explain the different expression profiles of resident microglia and infiltrating macrophages in physiological contexts and in brain malignancies. The first hypothesis is based on their distinct microenvironments of origin that facilitate and direct their differentiation [52, 53]. This leads to a specific expression profile of tumor-associated microglia compared with infiltrating macrophages. Specifically, their profile is enriched in cytokines, chemokines and complement components, and is correlated with antigen presentation and immune suppression functions. The second hypothesis is based on specific markers expressed by differentiated microglia compared with infiltrating macrophages. These markers include transmembrane protein 119 (TMEM119, a homeostatic microglia marker), P2Y12 (expressed by human microglia during development), Sal-like 1 (a transcriptional regulator that defines microglia identity and function), and sialic acid-binding immunoglobulin-type lectin (a specific microglia activation marker involved in tumor recognition and engulfment) [9].

The major challenge in these studies is the lack of specific, stable and standardized experimental systems. Consequently, the microglia heterogeneity and functional diversity, the contribution of infiltrating macrophages, and the specific roles of all these different subpopulations in healthy and disease/metastatic brain cannot be completely understood using classical methods and concepts.

## Microglia heterogeneity: the single-cell omic point of view

**Fig. 2:**
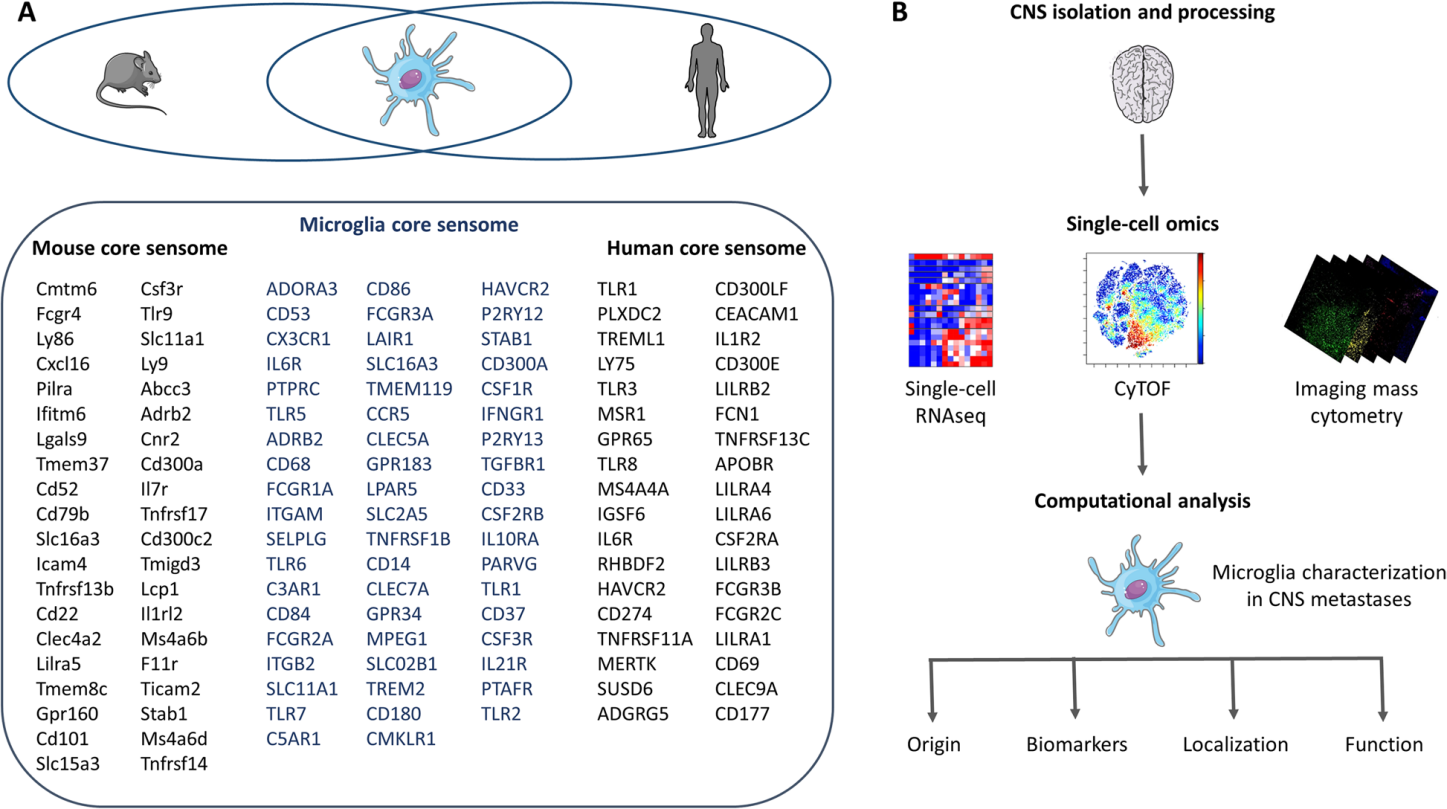
Microglia heterogeneity, insights from single-cell omics. **A** Overview of the similarities and differences between the mouse and human microglia sensomes. The microglia core sensome defines a set of genes that are expressed in both human and mouse microglia sensomes. **B** Schematic illustration of single-cell omic techniques (single-cell RNA-seq, CyTOF, and imaging mass cytometry) to obtain a high-resolution description of microglia in metastases to the brain in function of their origin, biomarkers, location, and function

Recent single-cell studies have provided new insights into microglia diversity. For instance, cytometry by time of flight (CyTOF), which is based on elemental metal isotopes conjugated to monoclonal antibodies, can simultaneously evaluate more than 40 parameters in individual cells with minimum overlap among channels [54]. In addition, single-cell RNA sequencing (scRNA-seq) allows determining the transcriptome profile of thousands of individual cells at a fixed point in time and in different conditions (health and disease) [55, 56] (Fig. 2).

### The microglia core sensome

Microglial cells constantly sense changes in the environment and adapt to them. This is possible because they express a specific set of genes that are called the microglia sensome and encode proteins that sense extracellular signals (e.g., purinergic receptors) (Fig. 2A). As first defined by Hickman et al., the microglia sensome is composed of the top 100 genes, mainly receptors, that are highly expressed on the microglial cell surface and that are involved in sensing potential pathological conditions. Abels et al., applied the approach developed by Hickman et al. to study the microglia sensome in other scRNA-seq datasets (i.e., data by Gosselin et al., [57] for the mouse microglia sensome and data by Gosselin et al., and Galatro et al., [57, 58] for the human microglia sensome). This allowed them to provide new transcriptome information on microglia and to identify similarity and differences between the murine and human sensomes. They found a significant overlap, including 57 genes that are highly expressed in both species and that they called the microglia core sensome (Fig. 2A). These genes are present in at least 75% of all analyzed microglia sensomes. To determine the microglia core sensome function, these 57 genes were ordered in eight different groups in function of their differential expression between microglia and cortex using the expression data from the study by Gosselin et al. [57]. These gene groups included purinergic and related receptors, cytokine receptors, chemokine and related receptors, Fc receptors, pattern recognition and related receptors, extracellular matrix receptors, endogenous ligands receptors/sensors and transporters, proteins involved in cell–cell interactions, and potential sensors without known ligands. Next, analysis of the specific ligands recognized by the identified sensome genes showed an overlap between the human and mouse ligands. This suggests that the mouse and human microglia can sense the same ligand groups. Then, the ligands recognized by the sensome receptors were classified in different ligand groups (i.e., glycoproteins, cytokines, immunoglobulins, amino acids, carbohydrates, electrolytes, lipopeptides, chemokines, neuraminic acids, nucleic acids, receptors, lipids, fatty acids, leukotrienes, hormones, steroids, and phospholipids). These ligands are involved in the most important physiological pathways necessary for cell function (this will be discussed in more details further in this section). If deregulated, these molecules can contribute also to brain tumor and metastasis development. For instance, it has been shown that cytokines, chemokines and their receptors, and TLRs (e.g. IL-6, IL-6R, CSF1R, CX3CR1, TLR3) are directly or indirectly linked to brain injuries, tumorigenesis, and metastasis [59–63]. Compared with the mouse sensome, the human core sensome includes a higher number of genes that encode extracellular matrix receptors, endogenous ligand receptors, sensors, and transporters.

Additional datasets were included to test the impact of this core sensome on CNS disorders (such as Alzheimer’s disease and amyotrophic lateral sclerosis) and during aging. Similar changes were identified in different datasets concerning the same disease or condition. Importantly, in conditions of brain damage, the microglia sensome was deregulated. In human microglia datasets, different microglia clusters were identified. They were characterized by the expression of CCL2, CCL4, EGR2 and EGR3, suggesting a more activated state of microglia. This might be due to environmental factors and to epigenetic differences between human and mouse microglia. Differences were detected also in genes involved in phagocytosis, complement, and susceptibility to neurodegenerative diseases [64].

Among the genes identified in the human core sensome, some might have a role in brain metastasis. For instance, IL-6 trans-signaling via the soluble IL-6 receptor (IL-6R) is crucial for microglia repopulation to robustly support neurogenesis, specifically by enhancing the survival of newborn neurons that directly support cognitive functions. This neuroprotective and pro-regenerative microglia phenotype can contribute to repair brain injuries and fight diseases, such as brain metastasis [63]. On the other hand, PD-L1 (CD274) has a pro-metastatic role in brain metastasis. It has been reported that recurrent brain metastases after radio-immunotherapy are partly due to the accumulation of PD-L1^+^ myeloid cells. Their presence indicates the establishment of an immune suppressive environment that counteracts the re-activated T-cell responses [65]. In addition, TLRs are transmembrane components that in physiological conditions sense danger signals, connect the innate (e.g., microglia) and adaptive immune systems (e.g., T cells) and contribute to tissue homeostasis. However, in cancer, TLR roles are contradictory depending on the cancer type/stage and immune microenvironment context. For instance, before metastasis initiation, TLR3 signaling promotes tumor cell death in breast and lung cancer and also in head and neck squamous cell carcinoma. TLR3 stimulation (e.g., by interferon type I signaling) results in cancer cell apoptosis in human and mouse models, or in the suppression of cancer cell migration, depending on the tumor stage. However, after the metastatic process initiation, TLR3 activation enhances tumor migration [61, 66, 67].

Moreover, microglia phagocytic activity, which is crucial for tumor and metastatic cell clearance, relies on specific receptors expressed on the cell surface (e.g., TLRs, and triggering receptor expressed on myeloid cells 2 (TREM-2)) and their downstream signaling pathways (e.g., NF-kB and IRF3, 4, 5, 7 and 8) [61, 68]. Changes in the microglia phagocytic state (e.g., increase in cell body size and decrease in process length) and increased microglia abundance in hippocampus could lead to abnormal brain pathologies. For instance, in a mouse model of Parkinson’s disease, increased microglial phagocytic activity and cell density induce synapse loss and upregulation of CSF1R and CSF2RA (microglia proliferation), CD68, ICAM1, and ICAM2 (microglial cell engulfment), and IL-6, IL-1β, CD11b, and TNFα (pro-inflammation molecules) in hippocampus [60]. Similarly, in humans, deregulation of CSF2RA (included in the human core sensome) might abnormally increase microglia phagocytosis and proliferation, thus leading to an exacerbation of chronic inflammation-associated brain metastasis.

Lastly, cell migration-inducing and hyaluronan-binding protein (CEMIP; not listed in the microglia sensome genes) is elevated in tumor tissues and exosomes from patients with brain metastases and predicts brain metastasis progression and decreased patient survival. Uptake of CEMIP^+^ exosomes by brain endothelial and microglial cells induces inflammation in the perivascular niche by upregulating TNF, and CCL/CXCL cytokines, known to promote brain vascular remodeling and metastasis [23].

Altogether, a decrease in sensome component expression could be associated with neurodegenerative disease development or tumor growth [69–72] and even with brain metastases. Importantly, when translating mouse results to humans, the similarities and differences between these species must be taken into account.

### Single-cell analyses of microglia in CNS metastases

Recently, in a xenograft lung-to-brain metastasis model, bulk and scRNA-seq data confirmed the functional heterogeneity of microglia and infiltrating macrophages, based on their origin. This suggests that several immune subsets coexist in the same diseased brain, but they exhibit different functions [18, 19, 73]. Other scRNA-seq analyses performed in primary brain tumor samples shed light on the phenotypic heterogeneity of brain tumor-infiltrating microglia. For instance, the first scRNA-seq analysis of tumor-infiltrating myeloid cells in dehydrogenase (IDH)-mutant adult glioblastoma samples found a microglia to macrophage-like cell phenotypic spectrum based on the gradual expression of their markers [74]. Using marker genes identified in murine glioma models, another scRNA-seq analysis described different signatures of microglia and macrophage subpopulations with a predominance of the M2 phenotype [75]. Moreover, using a multimodal single-cell analysis of the tumor microenvironment, Guldner et al. identified a heterogeneous, but spatially defined CNS myeloid response during brain metastasis growth, mostly promoted by microglia with a typical signature in which the homeostatic markers CX3CR1 and TMEM119 are downregulated. This leads to the enrichment of the interferon response and to CXCL10 upregulation that promote the pro-metastatic state maintenance and the immune suppressive niche via the recruitment of resident VISTA^hi^ and PD-L1^+^ myeloid cells to the metastatic site [76]. This spatial transcriptomic study allowed exploring the spatial location of microglia at high resolution in the context of brain metastases. Using a human brain metastasis tissue array, hypertrophic Iba1^+^ myeloid cells (first identified as microglia) were observed in the periphery of and within the human brain metastasis samples, independently of the primary tumor origin. The spatially defined morphological patterns of Iba1^+^ myeloid cells in brain metastases was also confirmed in a mouse model of brain metastases, irrespective of the brain topography and disease stage. Compared with control (healthy) brain, most Iba^+^ myeloid cells in brain metastases were hypertrophic with enlarged cell bodies and reduced protrusion features, indicating activation and a potent response. Principal component analysis of eight morphological features correlated with the distance between microglia and tumors (body volume, cell volume, distance to the nearest cell, number of protrusions, roundness, protrusion volume, protrusion width, total protrusion length) defined morphology scores that differentiated two distinct groups: naive myeloid cells with low morphology scores and brain metastasis-associated myeloid cells with higher morphology scores. Morphology scores increased in myeloid cells close to the brain metastatic lesion borders and were highest in myeloid cells within these lesions, suggesting their activation [76, 77].

In addition, single-cell proteomics and protein expression of human microglia have been evaluated by CyTOF that has larger panels compared with flow cytometry. Recently, CyTOF analysis of 74 immune cell parameters to describe leukocytes in the microenvironment of human glioma and brain metastases showed a clear distinction between glioma and brain metastasis samples. The glioma microenvironment presented predominantly reactive microglia. Conversely, tissue-invading leukocytes accumulated in brain metastases, with a preferential localization of infiltrating macrophages within the tumor core and of microglia in the tumor periphery [78] (Fig. 2B). Thorough investigations are needed to determine whether this regional specificity reflects a site-specific function or a particular vulnerability. Altogether, single-cell omics have given a clearer and at higher resolution picture of microglia functional and regional specialization in the context of brain tumors and metastases to the CNS.

## Microglia: a key therapeutic target for the management of metastases to the CNS?

**Fig. 3:**
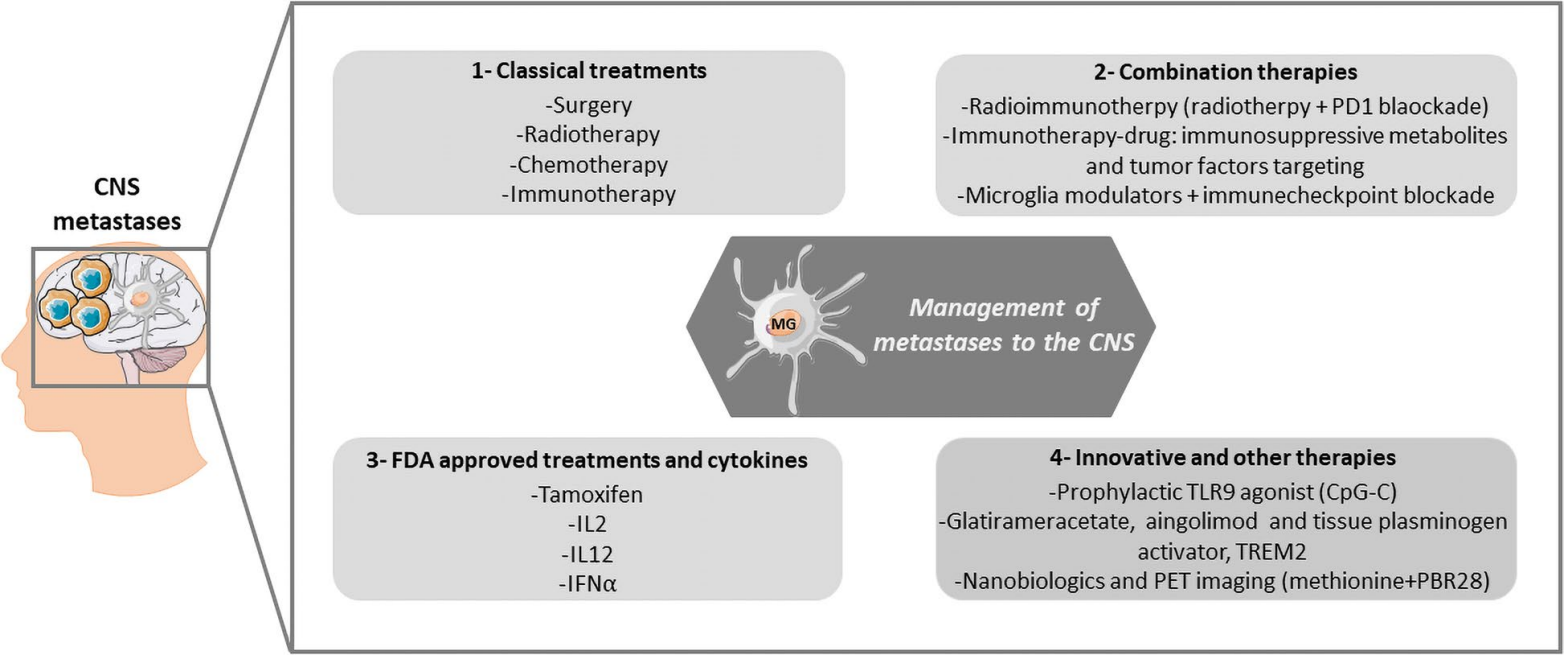
Management of metastases to the CNS. At late progression stage, some cancers will metastasize to the CNS where microglia (innate immune cells) and tumor cells tightly interact. Due to their crucial function and localization in the brain, microglia are a key therapeutic target in the management of patients with metastases in the CNS. Several therapies have been developed (or are currently being investigated) to treat metastases to the CNS, including: 1) classical treatments that minimally improve patient survival and are not curative; 2) combination therapies that may have a significant impact on the disease outcome (e.g., increased survival) by controlling both tumor cells and its microenvironment (e.g., microglia); 3) some FDA-approved therapeutics (e.g., tamoxifen and pro-inflammatory cytokines) can re-establish the anti-tumor function of microglia by skewing their M2 phenotype; and 4) innovative therapies that are currently under development or tested: a prophylactic treatment with a TLR9 agonist, other molecules (e.g., TREM2, glatirameracetate, aingolimod, tissue plasminogen activator) that are effective in other diseases (e.g., multiple sclerosis, stroke), PET imaging using methionine and PBR28 tracers, and nanobiologics for drug delivery and specific microglia targeting

Most cancers metastasize to the brain at late stages. Although several treatments (surgery, radiotherapy, targeted therapy, immunotherapy, and chemotherapy) (Fig. 3) have improved patient survival, metastasis incidence is not decreasing because their early detection is still difficult.

Due to the complex tumor cell-microglia interactions during metastasis formation in the brain, in vitro assays have limitations, including the fact that they cannot completely mimic the tumor microenvironment. Animal models are more appropriate because they allow studying the anatomical barriers (e.g. BBB), the crosstalk between cancer cells and microenvironment cells (e.g. microglia), and their responses in the specific context of metastases to the CNS. Patient-derived xenografts (PDX) are currently the best models. For instance, the PDX-based model of brain metastases from breast cancer developed and characterized by Contreras-Zarate et al., is a relevant tool to study brain colonization by human tumor cells. As PDX-based models maintain the molecular features and the heterogeneous nature of the patient tumor, they have a tremendous advantage compared with other mouse models of metastases, especially for evaluating potential therapeutic agents and identifying therapeutic targets [79].

Microglia, the brain immune resident cells, are strongly involved in brain metastasis development (see previous sections) and therefore, they are potential therapeutic targets. For instance, a study using an experimental breast-to-brain metastasis model showed that radiotherapy sensitizes brain metastases to immune checkpoint blockade. Radio-immunotherapy based on whole brain radiotherapy (2 Gy on five consecutive days) combined with immune checkpoint blockade (250 µg of anti-PD1 antibodies every third day starting with the first radiation dose) improved the T-cell anti-tumor response. The short-term efficacy of this therapy is explained by the re-establishment of the immune suppressive microenvironment due to post-treatment accumulation of PD-L1^+^ myeloid cells (microglia and infiltrating macrophages). This confirmed the crucial role of microglia immune suppressive function in brain metastases [65]. The brain metastatic lesions response to immunotherapy could be improved by combination with other drugs that target, for example, immunosuppressive metabolites produced by microglia. Indeed, the altered metabolism found in brain metastases has immunosuppressive functions [80–82]. Microglia and TAMs switch their metabolism to increased aerobic glycolysis. This results in increased lactate production and promotes the synthesis of immunosuppressive and tumor-promoting factors [83]. Moreover, microglial cells can respond to hypoxia and tumor metabolites by secreting immunosuppressive cytokines [84] Interestingly, it has been reported that microglia from brain metastatic lesions of patients with melanoma produce immunosuppressive metabolites, such as indolamine 2,3-dioxygenase [85].

In other mouse models, prophylactic treatment with CpG-C, a TLR9 agonist, significantly decreased brain metastasis development. In mice treated with CpG-C, activation of the microglia anti-tumor function led to the death and phagocytosis of tumor cells during the early stages of CNS invasion. These results suggest that this preventive approach could be advantageous for patients at high risk of CNS metastases. In addition, in vitro*,* in microglia-tumor cell co-cultures incubated with CpG-C, the enhanced phagocytic capacity and overall ameliorated functions of microglia induced an anti-tumor cytotoxic response. Interestingly, microglia depletion or inhibition abolished the favorable effect of CpG-C [59]. In a mouse model of brain metastases from estrogen receptor-deficient breast cancer, tamoxifen (an FDA-approved drug to block estrogen signaling) suppresses brain metastasis development by skewing M2 microglia polarization and by enhancing their anti-tumor phagocytic activity [86]. Moreover, some other FDA-approved drugs or molecules (e.g. glatirameracetate, aingolimod, tissue plasminogen activator) used for the treatment of specific diseases (e.g. multiple sclerosis, stroke) have shown promising effects on microglia function or polarization. Therefore, it could be worth to test their effect on microglia in metastatic disease [87, 88].

Anti-tumorigenic cytokines also have been evaluated in breast cancer brain metastasis models (EO771 breast cancer cells injected into the right frontal lobes of C57BL/6 mice) as immunogenic therapies. For instance, a cell therapy based on allogeneic fibroblasts modified to produce IL-2 significantly enhanced the overall survival in these mice [89]. In patients with metastatic melanoma, treatments based on high doses of pro-inflammatory cytokines, such as IL-2, IL-12 and IFN⍺, decrease the disease burden, but display modest anti-tumor activity [89–91].

Interestingly, Butovsky’s group identified a specific apolipoprotein E (APOE)-dependent molecular signature in microglia from mouse models of multiple amyotrophic lateral sclerosis and Alzheimer’s disease. This APOE pathway is induced by triggering receptor expressed on myeloid cells 2 (TREM2) that mediates the homeostasis-to-neurodegenerative switch in the microglia phenotype. Targeting the TREM2-APOE pathway restored the physiological microglia balance [92]. This study suggests that this pathway is a crucial regulator of microglia functional phenotype in neurodegenerative diseases and might represent a novel therapeutic target to restore microglia homeostasis. It could be interesting to test the impact of this pathway to limit microglia anti-tumor function in the context of metastases to the brain.

A recent clinical trial on microglia targeting in patients with brain metastases (NCT02433171) assessed the potential of positron emission tomography (PET) imaging using two different PET tracers: methionine that is sensitive to the tumor metabolic activity (with high tumor-to-normal brain contrast), and PBR28 that is sensitive to inflammatory processes (binds to translocator proteins upregulated in activated microglia). Their aim was to improve the discrimination between tumor recurrence and radiation necrosis in patients with melanoma and lung cancer brain metastases after stereotactic radiosurgery. They concluded that the sequential use of these two PET tracers is safe and effective. Moreover, they found that methionine is a reliable marker of tumor recurrence, but PBR28 is not good for radiation necrosis detection. To improve diagnostic imaging, more studies are needed to determine the causes of post-radiation inflammation and identify specific markers of radiation necrosis [93]. Other clinical trials on brain metastases registered at the ClinicalTrials.gov site will not be discussed here because they do not specifically target microglia.

Importantly, when using/developing drugs to target myeloid cells (such as microglia) and/or their polarization, their side effects, their toxicity and the potential overstimulation of the immune system should be taken into account because they could hamper the long-term results. To overcome the problems linked to the drug systemic distribution, natural biomaterials (such as high-density lipoprotein nanoparticles that have proved their efficacy in targeting macrophages) could be an effective alternative approach to specifically deliver drugs to the target immune cells (e.g. microglia) or for imaging to characterize their functional and phenotypic specificity, and their spatial localization. Nanobiologics could minimize the toxicity of the therapeutic agent and offer the benefits of therapeutic targeting and controlled release of the active molecule [94–96]. In addition, nano-targeted delivery strategies could increase the active compound ability to cross the BBB, thus enhancing drug effectiveness.

The complex nature of CNS metastases is reflected by the very short patients’ survival (few months) that has not been improved by the currently available treatments. Immune checkpoint inhibitors, such as PD-1/CTLA-4 blockade, improve patient survival [97]. However, to significantly increase the overall response rate, therapies that combine microglia modulation and immune checkpoint inhibitors (against CTLA4, PD1 or PD-L1) are needed to reactivate the T-cell cytotoxic response.

Altogether, due to their strategic function and localization in the brain, microglia seem to be a key therapeutic target for metastases in the CNS. Therapies to abrogate or deplete macrophages and microglia in animal models of brain metastases (e.g. colony-stimulating factor 1 receptor inhibitors) are currently investigated. An initial tumor response has been reported; however, resistance and its underlying mechanisms also have been detected [98]. Future research on therapeutic strategies for brain metastases must also focus on approaches to reprogram microglia by inhibiting their pro-tumorigenic response and supporting their anti-tumorigenic role. The ultimate goal of these therapies will be to rescue the original anti-tumor functions of microglia in the brain and to re-establish the immune homeostasis that is crucial to block tumor cell homing and metastasis growth. For that, the mechanisms underlying primary tumor, brain metastases, and metastasis tropism to other sites must be better understood and the specific microglia characteristics must be better defined.

## Conclusion

The consequences of the metastatic cancer cell-microglia interaction depend on their molecular and cellular characteristics that will determine the metastatic niche. The multi-dimensional tumor microenvironment that includes microglia, infiltrating macrophages, and other cell types further complicates the genetic/epigenetic regulation of the cell crosstalk with tumors, thus increasing the risk of tumor progression and invasion [99, 100].

The basic binary M1-M2 classification failed to clearly differentiate microglia from infiltrating macrophages that share a similar transcriptional network, with various degrees of activation and localization. Microglia are in a microenvironment that contains other cell types constantly interacting with them. Therefore, the classification in M1- and M2-polarized microglial cells is limited because their activation/phenotypic status may not be the only way in which microglia influence tumor development and spreading.

The use of scRNA-seq and CyTOF single-cell technologies to study microglia has brought new insights into microglia biological heterogeneity during CNS tumor progression and metastasis formation and has shown that microglial cells can have beneficial, detrimental, and dispensable functions. However, several limitations must be addressed: the small number of samples, the brain tissue quality, and the lack of precise information on the tumor microenvironment composition. To overcome this latest limitation, imaging mass cytometry (IMC) would be an excellent strategy to investigate tissue samples because it combines immunohistochemistry and mass cytometry with the possibility of using more than 40 markers at the same time [101]. This will allow describing the morphological and spatiotemporal dynamics of cell populations and their interactions within their microenvironment. Currently IMC has been used only to study human microglia in multiple sclerosis, and more efforts are needed to exploit this new technology to investigate microglia and their spatial location at high resolution in brain metastases. All these omics methods will help to specifically study microglia function, and to address many important questions with the ultimate aim of fully exploiting microglial cells for cancer metastasis treatment (Table 1).

**Table 1:** Outstanding questions

1) Why do some tumors preferentially colonize specific organs (e.g. brain)?
2) Is the microglia response to infiltrating metastatic cancer cells specific to the tumor type?
3) Is the microglia pro-inflammatory signature an anti-tumor immune response, or is it exploited by metastatic tumor cells to promote their colonization and growth in brain?
4) What are the microglia long-term effects on the metastatic disease progression?
5) Does cytokine level influence differently microglia in patient with CNS metastases compared with patients with CNS inflammatory diseases?
6) How can metastatic cancer cells escape the microglia surveillance and colonize the CNS microenvironment?
7) How do microglia respond to immune checkpoint-targeted therapy?

To answer the question in the title, from our prospective, microglia are undoubtedly playing an important multi-function and context-dependent role in brain metastases. Therefore, they might act as scapegoat, saboteur, or even something else.

## Data Availability

Not applicable.

## Abbreviations

ANG2: Angioprotein-2
BBB: Blood brain barrier
CEMIP: Cell migration-inducing and hyaluronan-binding protein
CNS: Central nervous system
CSFR: Colony-stimulating factor receptor
CXCL12: C-X-C motif chemokine ligand 12
CyTOF: Cytometry by time of flight
GDF-15: Growth differentiation factor 15
GM-CSF: Granulocyte–macrophage colony-stimulating factor
IMC: Imaging mass cytometry
LM: Leptomeningeal metastasis
MHC: Major histocompatibility complex
MMP: Matrix metalloprotease
PDX: Patient-derived xenografts
PET: Positron emission tomography
PI3K: Phosphoinositide 3-kinase
scRNA-seq: Single-cell RNA sequencing
SDF1: Stromal cell-derived factor1
STAT: Signal transducer and activator of transcription
TAMs: Tumor-associated macrophages
TGF: Transforming growth factor
TLR: Toll-like receptor
TMEM119: Transmembrane protein 119
TNF: Tumor necrosis factor
TREM2: Triggering receptor expressed on myeloid cells 2

## References

[1] Valiente M, Ahluwalia MS, Boire A (2018). The Evolving Landscape of Brain Metastasis. Trends in cancer.

[2] Sun L, Kees T, Almeida AS (2021). Activating a collaborative innate-adaptive immune response to control metastasis. Cancer Cell..

[3] Cagney DN, Martin AM, Catalano PJ (2017). Incidence and prognosis of patients with brain metastases at diagnosis of systemic malignancy: a population-based study. Neuro Oncol.

[4] Ostrom QT, Wright CH, Barnholtz-Sloan JS (2018). Brain metastases: epidemiology. Handb Clin Neurol.

[5] Quail DF, Joyce JA (2017). The Microenvironmental Landscape of Brain Tumors. Cancer Cell.

[6] Peinado H, Zhang H, Matei IR (2017). Pre-metastatic niches: organ-specific homes for metastases. Nat Rev.

[7] Sevenich L, Bowman RL, Mason SD (2014). Analysis of tumour- and stroma-supplied proteolytic networks reveals a brain-metastasis-promoting role for cathepsin S. Nat Cell Biol.

[8] Achrol AS, Rennert RC, Anders C (2019). Brain metastases Nat Rev Dis Primers.

[9] Schulz M, Salamero-Boix A, Niesel K, Alekseeva T, Sevenich L (2019). Microenvironmental Regulation of Tumor Progression and Therapeutic Response in Brain Metastasis. Front Immunol.

[10] Pukrop T, Dehghani F, Chuang HN (2010). Microglia promote colonization of brain tissue by breast cancer cells in a Wnt-dependent way. Glia.

[11] Winkler F (2015). The brain metastatic niche. J Mol Med (Berl).

[12] Ginhoux F, Greter M, Leboeuf M (2010). Fate mapping analysis reveals that adult microglia derive from primitive macrophages. Science..

[13] Gomez Perdiguero E, Klapproth K, Schulz C (2015). Tissue-resident macrophages originate from yolk-sac-derived erythro-myeloid progenitors. Nature.

[14] Kierdorf K, Erny D, Goldmann T (2013). Microglia emerge from erythromyeloid precursors via Pu.1- and Irf8-dependent pathways. Nature Neurosci..

[15] Butovsky O, Weiner HL (2018). Microglial signatures and their role in health and disease. Nat Rev Neurosci.

[16] Chuang HN, van Rossum D, Sieger D (2013). Carcinoma cells misuse the host tissue damage response to invade the brain. Glia.

[17] Qiao S, Qian Y, Xu G, Luo Q, Zhang Z (2019). Long-term characterization of activated microglia/macrophages facilitating the development of experimental brain metastasis through intravital microscopic imaging. J Neuroinflammation.

[18] Deczkowska A, Keren-Shaul H, Weiner A, Colonna M, Schwartz M, Amit I (2018). Disease-Associated Microglia: A Universal Immune Sensor of Neurodegeneration. Cell.

[19] Schulz M, Michels B, Niesel K (2020). Cellular and Molecular Changes of Brain Metastases-Associated Myeloid Cells during Disease Progression and Therapeutic Response. iScience.

[20] Fares J, Fares MY, Khachfe HH, Salhab HA, Fares Y (2020). Molecular principles of metastasis: a hallmark of cancer revisited. Signal Transduct Target Ther.

[21] Gan DX, Wang YB, He MY (2020). Lung Cancer Cells-Controlled Dkk-1 Production in Brain Metastatic Cascade Drive Microglia to Acquire a Pro-tumorigenic Phenotype. Front Cell Dev Biol.

[22] Li YD, Lamano JB, Lamano JB (2019). Tumor-induced peripheral immunosuppression promotes brain metastasis in patients with non-small cell lung cancer. Cancer Immunol Immunother.

[23] Rodrigues G, Hoshino A, Kenific CM (2019). Tumour exosomal CEMIP protein promotes cancer cell colonization in brain metastasis. Nat Cell Biol.

[24] Wu SY, Xing F, Sharma S, et al. Nicotine promotes brain metastasis by polarizing microglia and suppressing innate immune function. J Exp Med. 2020;217(8):e20191131. 10.1084/jem.20191131 PMC7398164 32496556

[25] Salmaggi A, Maderna E, Calatozzolo C (2009). CXCL12, CXCR4 and CXCR7 expression in brain metastases. Cancer Biol Ther.

[26] Vogel DY, Kooij G, Heijnen PD (2015). GM-CSF promotes migration of human monocytes across the blood brain barrier. Eur J Immunol.

[27] Lorger M, Felding-Habermann B (2010). Capturing changes in the brain microenvironment during initial steps of breast cancer brain metastasis. Am J Pathol.

[28] Xing F, Liu Y, Wu SY (2018). Loss of XIST in Breast Cancer Activates MSN-c-Met and Reprograms Microglia via Exosomal miRNA to Promote Brain Metastasis. Can Res.

[29] Andreou KE, Soto MS, Allen D (2017). Anti-inflammatory Microglia/Macrophages As a Potential Therapeutic Target in Brain Metastasis. Front Oncol.

[30] Simon A, Yang M, Marrison JL (2020). Metastatic breast cancer cells induce altered microglial morphology and electrical excitability in vivo. J Neuroinflammation.

[31] Priego N, Zhu L, Monteiro C (2018). STAT3 labels a subpopulation of reactive astrocytes required for brain metastasis. Nat Med.

[32] Helfrich I, Edler L, Sucker A (2009). Angiopoietin-2 levels are associated with disease progression in metastatic malignant melanoma. Clin Cancer Res.

[33] Weide B, Schafer T, Martens A (2016). High GDF-15 Serum Levels Independently Correlate with Poorer Overall Survival of Patients with Tumor-Free Stage III and Unresectable Stage IV Melanoma. J Invest Dermatol.

[34] Izraely S, Ben-Menachem S, Sagi-Assif O (2019). The metastatic microenvironment: Melanoma-microglia cross-talk promotes the malignant phenotype of melanoma cells. Int J Cancer.

[35] Chen XW, Zhou SF (2015). Inflammation, cytokines, the IL-17/IL-6/STAT3/NF-kappaB axis, and tumorigenesis. Drug Des Dev Ther.

[36] Zhang C, Zhang F, Tsan R, Fidler IJ (2009). Transforming growth factor-beta2 is a molecular determinant for site-specific melanoma metastasis in the brain. Can Res.

[37] Moshe A, Izraely S, Sagi-Assif O (2018). Cystatin C takes part in melanoma-microglia cross-talk: possible implications for brain metastasis. Clin Exp Metas.

[38] Bienkowski M, Preusser M (2015). Prognostic role of tumour-infiltrating inflammatory cells in brain tumours: literature review. Curr Opin Neurol.

[39] He BP, Wang JJ, Zhang X, et al. Differential reactions of microglia to brain metastasis of lung cancer. Mol Med. 2006;12(7–8):161–70. 10.2119/2006-00033.He PMC1626596 17088948

[40] Kinjyo I, Bragin D, Grattan R, Winter SS, Wilson BS (2019). Leukemia-derived exosomes and cytokines pave the way for entry into the brain. J Leukoc Biol.

[41] Lu JQ, Menon S, Fong C, Lach B, Power C (2018). Tumor-to-Lesion Metastasis: Case Report of Carcinoma Metastasis to Multiple Sclerosis Lesion. World Neurosurg.

[42] Ellert-Miklaszewska A, Dabrowski M, Lipko M, Sliwa M, Maleszewska M, Kaminska B (2013). Molecular definition of the pro-tumorigenic phenotype of glioma-activated microglia. Glia.

[43] Gabrusiewicz K, Ellert-Miklaszewska A, Lipko M, Sielska M, Frankowska M, Kaminska B (2011). Characteristics of the alternative phenotype of microglia/macrophages and its modulation in experimental gliomas. PLoS ONE.

[44] Yu H, Pardoll D, Jove R (2009). STATs in cancer inflammation and immunity: a leading role for STAT3. Nat Rev Cancer.

[45] Feng X, Szulzewsky F, Yerevanian A (2015). Loss of CX3CR1 increases accumulation of inflammatory monocytes and promotes gliomagenesis. Oncotarget.

[46] Juedes AE, Ruddle NH (2001). Resident and infiltrating central nervous system APCs regulate the emergence and resolution of experimental autoimmune encephalomyelitis. J Immunol.

[47] Ulvestad E, Williams K, Bjerkvig R, Tiekotter K, Antel J, Matre R (1994). Human microglial cells have phenotypic and functional characteristics in common with both macrophages and dendritic antigen-presenting cells. J Leukoc Biol.

[48] Zhang B, Wei YZ, Wang GQ, Li DD, Shi JS, Zhang F (2019). Targeting MAPK Pathways by Naringenin Modulates Microglia M1/M2 Polarization in Lipopolysaccharide-Stimulated Cultures. Front Cell Neurosci.

[49] Karnevi E, Andersson R, Rosendahl AH (2014). Tumour-educated macrophages display a mixed polarisation and enhance pancreatic cancer cell invasion. Immunol Cell Biol.

[50] Kortylewski M, Kujawski M, Wang T (2005). Inhibiting Stat3 signaling in the hematopoietic system elicits multicomponent antitumor immunity. Nat Med.

[51] Aguzzi A, Barres BA, Bennett ML (2013). Microglia: scapegoat, saboteur, or something else?. Science..

[52] Jordao MJC, Sankowski R, Brendecke SM, et al. Single-cell profiling identifies myeloid cell subsets with distinct fates during neuroinflammation. Science. 2019;363(6425):eaat7554. 10.1126/science.aat7554 30679343

[53] Van Hove H, Martens L, Scheyltjens I (2019). A single-cell atlas of mouse brain macrophages reveals unique transcriptional identities shaped by ontogeny and tissue environment. Nat Neurosci.

[54] Fernandez-Zapata C, Leman JKH, Priller J, Bottcher C (2020). The use and limitations of single-cell mass cytometry for studying human microglia function. Brain pathology (Zurich, Switzerland).

[55] Abels ER, Nieland L, Hickman S, Broekman MLD, El Khoury J, Maas SLN. Comparative Analysis Identifies Similarities between the Human and Murine Microglial Sensomes. Int J Mol Sci. 2021;22(3):1495. 10.3390/ijms22031495 PMC7867338 33540859

[56] Ochocka N, Kaminska B. Microglia Diversity in Healthy and Diseased Brain: Insights from Single-Cell Omics. Int J Mol Sci. 2021;22(6):3027. 10.3390/ijms22063027 PMC8002227 33809675

[57] Gosselin D, Skola D, Coufal NG, et al. An environment-dependent transcriptional network specifies human microglia identity. Science. 2017;356(6344):eaal3222. 10.1126/science.aal3222 PMC5858585 28546318

[58] Galatro TF, Holtman IR, Lerario AM (2017). Transcriptomic analysis of purified human cortical microglia reveals age-associated changes. Nat Neurosci.

[59] Benbenishty A, Gadrich M, Cottarelli A (2019). Prophylactic TLR9 stimulation reduces brain metastasis through microglia activation. PLoS Biol.

[60] Ding W, Lin H, Hong X, Ji D, Wu F. Poloxamer 188-mediated anti-inflammatory effect rescues cognitive deficits in paraquat and maneb-induced mouse model of Parkinson’s disease. Toxicology. 2020;436:152437. 10.1016/j.tox.2020.152437 32169474

[61] Anwar MA, Eskian M, Keshavarz-Fathi M, Choi S, Rezaei N, KhajehAlizadeh Attar M (2018). Basic understanding and therapeutic approaches to target toll-like receptors in cancerous microenvironment and metastasis. Med Res Rev..

[62] Pyonteck SM, Akkari L, Schuhmacher AJ (2013). CSF-1R inhibition alters macrophage polarization and blocks glioma progression. Nat Med.

[63] Willis EF, MacDonald KPA, Nguyen QH (2020). Repopulating Microglia Promote Brain Repair in an IL-6-Dependent Manner. Cell.

[64] Geirsdottir L, David E, Keren-Shaul H (2019). Cross-Species Single-Cell Analysis Reveals Divergence of the Primate Microglia Program. Cell.

[65] Niesel K, Schulz M, Anthes J (2021). The immune suppressive microenvironment affects efficacy of radio-immunotherapy in brain metastasis. EMBO Mol Med.

[66] Cheng YS, Xu F (2010). Anticancer function of polyinosinic-polycytidylic acid. Cancer Biol Ther.

[67] Rydberg C, Mansson A, Uddman R, Riesbeck K, Cardell LO (2009). Toll-like receptor agonists induce inflammation and cell death in a model of head and neck squamous cell carcinomas. Immunology.

[68] Fu R, Shen Q, Xu P, Luo JJ, Tang Y (2014). Phagocytosis of microglia in the central nervous system diseases. Mol Neurobiol.

[69] Hickman SE, El Khoury J (2019). Analysis of the Microglial Sensome. Methods Mol Bio..

[70] Hickman SE, Kingery ND, Ohsumi TK (2013). The microglial sensome revealed by direct RNA sequencing. Nat Neurosci.

[71] Izzy S, Liu Q, Fang Z (2019). Time-Dependent Changes in Microglia Transcriptional Networks Following Traumatic Brain Injury. Front Cell Neurosci.

[72] Maas SLN, Abels ER, Van De Haar LL (2020). Glioblastoma hijacks microglial gene expression to support tumor growth. J Neuroinflammation.

[73] Mrdjen D, Pavlovic A, Hartmann FJ (2018). High-Dimensional Single-Cell Mapping of Central Nervous System Immune Cells Reveals Distinct Myeloid Subsets in Health, Aging, and Disease. Immunity.

[74] Venteicher AS, Tirosh I, Hebert C, et al. Decoupling genetics, lineages, and microenvironment in IDH-mutant gliomas by single-cell RNA-seq. Science. 2017;355(6332):eaai8478. 10.1126/science.aai8478 PMC5519096 28360267

[75] Muller S, Kohanbash G, Liu SJ (2017). Single-cell profiling of human gliomas reveals macrophage ontogeny as a basis for regional differences in macrophage activation in the tumor microenvironment. Genome Biol.

[76] Guldner IH, Wang Q, Yang L (2020). CNS-Native Myeloid Cells Drive Immune Suppression in the Brain Metastatic Niche through Cxcl10. Cell.

[77] Heindl S, Gesierich B, Benakis C, Llovera G, Duering M, Liesz A (2018). Automated Morphological Analysis of Microglia After Stroke. Front Cell Neurosci.

[78] Friebel E, Kapolou K, Unger S (2020). Single-Cell Mapping of Human Brain Cancer Reveals Tumor-Specific Instruction of Tissue-Invading Leukocytes. Cell.

[79] Contreras-Zarate MJ, Ormond DR, Gillen AE (2017). Development of Novel Patient-Derived Xenografts from Breast Cancer Brain Metastases. Front Oncol.

[80] Fischer GM, Jalali A, Kircher DA (2019). Molecular Profiling Reveals Unique Immune and Metabolic Features of Melanoma Brain Metastases. Cancer Discov.

[81] Marullo R, Castro M, Yomtoubian S (2021). The metabolic adaptation evoked by arginine enhances the effect of radiation in brain metastases. Science advances.

[82] Tyagi A, Wu SY, Watabe K (2022). Metabolism in the progression and metastasis of brain tumors. Cancer Lett.

[83] Longhitano L, Vicario N, Forte S, et al. Lactate modulates microglia polarization via IGFBP6 expression and remodels tumor microenvironment in glioblastoma. Cancer Immunol Immunother. 2022. 10.1007/s00262-022-03215-3 PMC9813126 35654889

[84] Colegio OR, Chu NQ, Szabo AL (2014). Functional polarization of tumour-associated macrophages by tumour-derived lactic acid. Nature.

[85] Herrera-Rios D, Mughal SS, Teuber-Hanselmann S (2020). Macrophages/Microglia Represent the Major Source of Indolamine 2,3-Dioxygenase Expression in Melanoma Metastases of the Brain. Front Immunol.

[86] Wu SY, Sharma S, Wu K (2021). Tamoxifen suppresses brain metastasis of estrogen receptor-deficient breast cancer by skewing microglia polarization and enhancing their immune functions. Breast Cancer Res.

[87] Nally FK, De Santi C, McCoy CE. Nanomodulation of Macrophages in Multiple Sclerosis. Cells. 2019;8(6):543. 10.3390/cells8060543 PMC6628349 31195710

[88] Gravanis I, Tsirka SE (2005). Tissue plasminogen activator and glial function. Glia.

[89] Deshmukh P, Glick RP, Lichtor T, Moser R, Cohen EP (2001). Immunogene therapy with interleukin-2-secreting fibroblasts for intracerebrally metastasizing breast cancer in mice. J Neurosurg.

[90] Gonzalez Cao M, Malvehy J, Marti R (2006). Biochemotherapy with temozolomide, cisplatin, vinblastine, subcutaneous interleukin-2 and interferon-alpha in patients with metastatic melanoma. Melanoma Res.

[91] Triozzi PL, Strong TV, Bucy RP (2005). Intratumoral administration of a recombinant canarypox virus expressing interleukin 12 in patients with metastatic melanoma. Hum Gene Ther.

[92] Krasemann S, Madore C, Cialic R (2017). The TREM2-APOE Pathway Drives the Transcriptional Phenotype of Dysfunctional Microglia in Neurodegenerative Diseases. Immunity.

[93] Tran TT, Gallezot JD, Jilaveanu LB (2020). [(11)C]Methionine and [(11)C]PBR28 as PET Imaging Tracers to Differentiate Metastatic Tumor Recurrence or Radiation Necrosis. Mol Imaging.

[94] Ochando J, Braza MS (2017). Nanoparticle-Based Modulation and Monitoring of Antigen-Presenting Cells in Organ Transplantation. Front Immunol.

[95] Fay F, Hansen L, Hectors S (2017). Investigating the Cellular Specificity in Tumors of a Surface-Converting Nanoparticle by Multimodal Imaging. Bioconjug Chem.

[96] Braza MS, van Leent MMT, Lameijer M (2018). Inhibiting Inflammation with Myeloid Cell-Specific Nanobiologics Promotes Organ Transplant Acceptance. Immunity.

[97] Iorgulescu JB, Harary M, Zogg CK (2018). Improved Risk-Adjusted Survival for Melanoma Brain Metastases in the Era of Checkpoint Blockade Immunotherapies: Results from a National Cohort. Cancer Immunol Res.

[98] Klemm F, Mockl A, Salamero-Boix A (2021). Compensatory CSF2-driven macrophage activation promotes adaptive resistance to CSF1R inhibition in breast-to-brain metastasis. Nature cancer.

[99] Ochando J, Braza MS (2017). T follicular helper cells: a potential therapeutic target in follicular lymphoma. Oncotarget.

[100] Amin R, Braza MS (2022). The follicular lymphoma epigenome regulates its microenvironment. J Exp Clin Cancer Res.

[101] Giesen C, Wang HA, Schapiro D (2014). Highly multiplexed imaging of tumor tissues with subcellular resolution by mass cytometry. Nat Methods.

